# Agro-Climatic Suitability Evaluation for Saffron Production in Areas of Western Himalaya

**DOI:** 10.3389/fpls.2021.657819

**Published:** 2021-03-15

**Authors:** Deepak Kothari, Meenakshi Thakur, Robin Joshi, Amit Kumar, Rakesh Kumar

**Affiliations:** ^1^Academy of Scientific and Innovative Research (AcSIR), Ghaziabad, India; ^2^Agrotechnology Division, CSIR-Institute of Himalayan Bioresource Technology (Council of Scientific and Industrial Research), Palampur, India; ^3^Biotechnology Division, CSIR-Institute of Himalayan Bioresource Technology (Council of Scientific and Industrial Research), Palampur, India; ^4^Environmental Technology Division, CSIR-Institute of Himalayan Bioresource Technology (Council of Scientific and Industrial Research), Palampur, India

**Keywords:** crocin, locations, picrocrocin, safranal, soil

## Abstract

Saffron (*Crocus sativus* L.) is an expensive spice crop cultivated successfully in Iran, Afghanistan, India, Greece, Morocco, Spain, and Italy. The present study was conducted during the periods 2018–2019 and 2019–2020 to evaluate the morphological, yield and quality parameters of saffron in six different regions of non-traditional areas of the western Himalayas. The two experimental factors were “year” and “location.” The experiment was conducted using a factorial randomized block design with three replications. Yield attributes, *viz.*, number of flowers, fresh flower yield, fresh stigma yield and dry stigma yield were significantly higher in location L_3_ compared to other geographical locations. Dry stigma yield in location L_3_ was higher by 50.0, 41.2, 33.3, 14.3, and 9.1% compared to locations L_6_, L_5_, L_1_, L_4_, and L_2_, respectively. These were characterized by the appropriate climatic conditions, *viz.*, high altitude, sandy-loam texture of the soil, optimum temperature, lesser relative humidity and total rainfall, demonstrating that it is possible to cultivate this spice even in non-traditional areas of the western Himalaya. Positive correlations were established for stigma yield with increased altitude and lesser rainfall. Secondary metabolites *viz.*, crocin and picrocrocin increased significantly with the increase in altitude; however, a reverse trend was recorded for safranal content. Total phenolics and flavonoids were significantly higher in the geographical location of Kinnaur, H.P. (L_1_) and Bharmour, H.P. (L_4_). In conclusion, the assessment of different geographical locations and soil types is particularly necessary to encourage saffron production and its qualitative traits. Based on current findings, saffron can be grown successfully in some non-traditional locations of the western Himalayan regions.

## Introduction

Saffron (*Crocus sativus* L.) “(family Iridaceae),” commonly known as red gold, is one of the most expensive and valuable spice crops in the world market (Gomez-Gomez et al., 2012). It is a triploid plant that is propagated through corms (Bayat et al., 2016). More than 418 tons/annum of saffron are produced worldwide from an area of 108,000 ha in Iran, 7,557 ha in Afghanistan, 3,674 ha in India, 1,000 ha in Greece, 850 ha in Morocco, 150 ha in Spain, 70 ha in Italy and 37 ha in France (Cardone et al., 2020a). Saffron flowers in the range of 75–100 provide 225–300 stigma threads, which produce only 0.5 g of dry stigma (Gohari et al., 2013). The crop price is very high and varies from 1,500 to 2,200 Euro/kg (Mykhailenko et al., 2020). Nowadays, saffron cultivation is gaining interest due to its major uses in industries, *viz*., the textile, dye, drug and culinary adjunct, food additive, coloring, and flavoring industries; furthermore, it has also gained interest for it’s pharmacological properties, e.g., antioxidant, antitumor activity, anticancer, and antimutagenic activity (Moradi Rikabad et al., 2019; Mykhailenko et al., 2020).

Altitude, soil characteristics, temperature, photoperiod, and topographical locations are the critical environmental parameters that affect saffron production (Siracusa et al., 2010; Rahimi et al., 2017; Cardone et al., 2020b). This spice crop grows well in friable, loose, low density, well-irrigated and well-drained clay calcareous soils with an optimum pH range between 6.8–7.8 and electrical conductivity (E.C.) below 2 dS m^–1^ (Zarghani et al., 2016). It is cultivated mainly in Iran, Afghanistan, Morocco, India, Spain, Greece, and Italy (Cardone et al., 2019). Previous studies suggest that 600 mm seasonal rainfall is almost sufficient for saffron cultivation under rainfed conditions, which can vary depending on soil characteristics and fertilization practices (Fallahi and Mahmoodi, 2018a,b). Gresta et al. (2009) reported the highest flower number and dry stigma yield when corms were planted with high density in sandy soil, while the highest stigma weight was obtained on sowing corms in clay soil with high density.

The quality of the saffron entirely depends on the environmental conditions, content and composition of secondary metabolites. The major marker compounds crocins, picrocrocin and safranal are responsible for the coloring, bittering and aromatic powers, respectively (Siracusa et al., 2010; Garcia-Rodriguez et al., 2017; Giorgi et al., 2017; Mykhailenko et al., 2020). Besides the significant amount of information that exists on aspects of physiological, morphological, genotype, chemical, and genetic diversity, there remains a lack of information on different environmental conditions that alter the yield and secondary metabolite profile of the saffron crop. In India, it is only cultivated in traditional areas, *viz.*, Pulwama and Kishtwar districts in Jammu and Kashmir, in silty clay loam textures with an electrical conductivity ranging from 0.09 to 0.30 dS m^–1^, pH between 6.3 and 8.3, calcium carbonate content of 4.61% and average organic carbon of 0.35% (Ganaie and Singh, 2019). Although many studies have been performed, the adaptability of this crop under diverse environmental conditions for studying different vegetative, qualitative and quantitative traits needs to be further investigated under the western Himalayan conditions (Cardone et al., 2020b).

To meet the increased demand for saffron, an attempt was made in India in Himachal Pradesh and Uttarakhand with the following objectives: (i) to identify the suitable locations for the cultivation of saffron in different regions of western Himalayas and (ii) to test the quality of the harvested spice using ultra pressure liquid chromatography (UPLC).

## Materials and Methods

### Experimental Site Description and Planting Material

**FIGURE 1:**
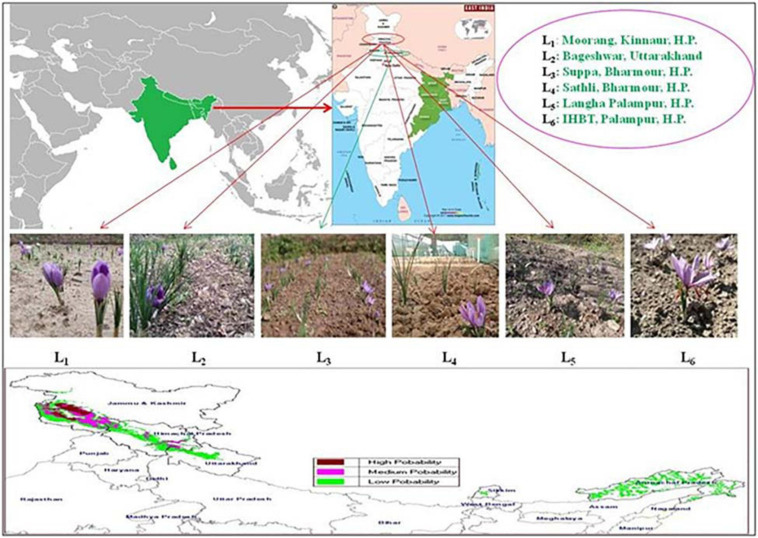
Identification of different locations through the MAXENT model.

The experiment was conducted during the years 2018–2019 and 2019–2020 at six geographical locations of different altitudinal zones of Himachal Pradesh and Uttarakhand, characterized by different environmental conditions. Potential locations for the experiment were identified with the maximum entropy (MAXENT) model on a map and depicted in Figure 1. The study sites were Moorang, Kinnaur, Himachal Pradesh (L_1_), Kapkote, Bageshwar, Uttarakhand (L_2_), Suppa, Bharmour, Himachal Pradesh (L_3_), Sathli, Bhamour, Himachal Pradesh (L_4_), Langha, Palampur, Himachal Pradesh (L_5_) and the Council of Scientific and Industrial Research-Institute of Himalayan Bioresource Technology, Palampur, Himachal Pradesh (L_6_). Geographic coordinates (latitude and longitude), altitude and an average of 2 years’ standard meteorological data (average minimum and maximum temperatures, relative humidity and total rainfall) during cultivation seasons from each site were extrapolated from meteorological stations nearest each sample site. Saffron corms were procured from Kishtwar, Jammu and Kashmir, India. The average size of each saffron corm was 10–15 g.

### Agronomic Management

At each location, the land was prepared by manual labor. Raised beds 8 m^2^ in size were prepared. Farmyard manure (25 t/ha) was added before plantation at each site and corms were treated with a fungicide solution to reduce fungal diseases. During September, corms were planted at different geographical locations; the plots were managed, and crop yield data were collected for 2 years. Saffron corms were planted at 12 cm depth with the spacing of 20 cm × 10 cm (row to row X plant to plant). Two factorial approaches were considered with 2 years and six planting sites in a factorial randomized block design (RBD). The experiment was executed in three replications, which were repeated for 2 years. Similar cultural practices designed by the Council of Scientific and Industrial Research-Institute of Himalayan Bioresource Technology, Palampur, Himachal Pradesh, India were implemented at different sites. Therefore, the differences between their performances are primarily due to different environmental conditions. The data on the number of flowers/m^2^, fresh flower yield (kg/ha), fresh stigma yield (kg/ha) and dry stigma yield (kg/ha) were recorded. Growth parameters, *viz.*, the number of leaves and leaf lengths, were recorded from January to February at all locations during its life cycle. During the flowering season, flowers were plucked daily at early hours in the morning before perianth openings to minimize the loss of volatile compounds of economic importance. After plucking, fresh stigmas were removed from the remaining flower immediately and were shade dried at room temperature.

### Quality Analysis

Saffron dry stigmas were stored in the dark inside closed glass jars at room temperature (18–22°C) until further analysis (Gresta et al., 2008). Dried saffron stigmas (50 mg) were crushed in a mortar pestle to form a powder. Saffron powder was extracted using 70% methanol, followed by 5 min of vortex mixing and centrifugation at 8,000 rpm at 4°C for 10 min. The final volume of the extract was made up to 5 mL, with 70% methanol, and the samples were kept at 4°C for further analysis (Joshi et al., 2015).

### Total Flavonoid Content

The flavonoid content of the saffron extract was estimated by using Joshi et al. (2015) method with minor alteration. The saffron methanol extract (1 mL) was diluted with distilled water, and then 5% of sodium nitrate was added to the solution. After 5 min., 3 mL of 10% aluminum chloride was added. After 6 min of incubation 2 mL of 1 M sodium hydroxide was added, and the sample was kept for 1-h incubation. Absorbance was taken at 510 nm wavelength against blank with a Shimadzu 8000 UV-Vis spectrophotometer. Total flavonoid content was expressed as mg/g equivalent of quercetin. Readings were taken in triplicate.

### Total Phenolic Content

Total phenolic content was calculated using the method of Kumar et al. (2020). In 1.5 mL Folin-Ciocalteu Reagent (1 F.C.: 9 Water), 300 μL of methanol extract was added and incubated at room temperature for 10 min in dark conditions (Kumar et al., 2020). After that 1.2 mL of 2.5% Na_2_CO_3_ solution was added and the mixture was kept idle for 1 h. Absorbance was taken at 765 nm using a Shimadzu 8000 UV-Vis spectrophotometer. Gallic acid was expressed in the total phenolic content as mg/g equivalent. Readings were taken in triplicates.

### Ultra Pressure Liquid Chromatography Analysis

Acquity UPLC-H Class System was used to analyze marker compounds, *viz.*, crocin, picrocrocin and safranal, with an auto sampler, binary solvent manager and a column heater equipped with a PDA detector (Joshi et al., 2015). For separation, the BEH C18 column (2.1 mm × 50 mm, 1.7 μm) was used. The column-heater was set at a temperature of 35°C with flow rate of 0.2 mL per min). Eluents A and B were 0.1% formic acid in water and 0.1% formic acid in acetonitrile, respectively. The non-linear separation gradient was 0–0.5 min (90% A), 4 min (50% A), 9 min (20% A), 10 min (10% A), 11 min (10% A), 12 min (90% A), and 15 min (90% A). One μL of the sample was injected for analysis. Set the eλ PDA detector at 354, 254, and 340 nm. The mean area of three replicate injections was considered for quantification against calibration curves for each standard (Joshi et al., 2015).

### Soil Analysis

Soil samples collected from each location were dried at room temperature and passed through a 30 mesh panel. The method of Mehlich No. 3 (Mehlich, 1984) was used to estimate available P_2_O_5_ and K_2_O. The Walkley and Black method was used to determine the organic carbon, and the macro Kjeldahl method was used for the analysis of available nitrogen content (Black, 1965). The hydrometer method and pH meter (Black, 1965) were used to determine the soil texture and pH of the soil.

### Statistical Analysis

Data collected on different yield and growth parameters of saffron was subjected to analysis of variance (ANOVA) in factorial randomized block design (RBD). Treatment variance was measured by the values of the least significant difference (LSD) at *P* = *0.05* and *P* = *0.01* by multiplying standard error of mean (SEM) values. For correlation studies, software XLSTAT 2017 was used to explore the relationship between growth and yield parameters. Past three software was used to analyze the principal component analysis (PCA). Performance measuring and ranking by main component analysis (PSR-PCA) provides a useful tool for evaluating performance under various factors (Coussement et al., 2016).

## Results and Discussion

### Climate and Soil Characteristics at Experimental Sites

Weather data of six different altitudinal locations is presented in Table 1. The highest altitude was observed for location L_1_ (2,591 m) followed by L_2_ (2,400 m), L_3_ (2,195 m), L_4_ (2,019 m), L_5_ (1,565 m) and L_6_ (1,472 m). This study identified and validated favorable environmental conditions under the western Himalayas for saffron cultivation through the MAXENT model. The altitude of selected locations varied from 1,472 to 2,591 m amsl. It is understood that saffron’s productivity and efficiency have a major impact on altitudinal variability (Mykhailenko et al., 2020). Throughout India, only the Kashmir valley represents one of the most significant saffron growing areas situated at an altitude of 1,585–1,677 m amsl under temperate climatic conditions (Ganaie and Singh, 2019).

**TABLE 1:** Location, geographical and climatic characteristics of the study area of saffron crop.

	**Location**			**Geographical coordinates**		**Altitude (m)**	**Average temperature (°C)**		**Total rainfall (mm)**	**Relative humidity (%)**
**Code**	**Site**	**District**	**State**	**Latitude**	**Longitude**		**Max.**	**Min.**		
L_1_	Moorang	Kinnaur	Himachal Pradesh	31°36′10″	78°27′04″	2,591	20.9	8.5	998.9	49.6
L_2_	Kapkote	Bageshwar	Uttarakhand	30°05′05′	79°53′58″	2,400	23.9	8.6	1,223.8	60.6
L_3_	Suppa, Bharmour	Chamba	Himachal Pradesh	32°26′47″	76°34′14″	2,195	28.2	16.5	742.7	38.3
L_4_	Sathli, Bharmour	Chamba	Himachal Pradesh	32°26′47″	76°34′14″	2,019	27.5	17.1	1,195.3	42.7
L_5_	Langha, Palampur	Kangra	Himachal Pradesh	32°07′49″	76°33′54″	1,565	26.2	15.7	2,254.8	46.2
L_6_	CSIR-IHBT Palampur	Kangra	Himachal Pradesh	32°06′29″	76°33′35″	1,472	27.8	17.7	2,193.1	46.8

The mean value of air temperature (maximum and minimum) was lowest in locations L_1_ (20.9°C) and L_2_ (23.9°C). In the case of maximum temperature, location L_3_ showed the highest temperature (28.2°C) followed by location L_6_ (27.8°C), L_4_ (27.5°C), and L_5_ (26.2°C). Location L_6_ showed the highest average range of minimum temperature (17.7°C) compared with other locations. During the crop period, total rainfall received was 998.9 mm in L_1_, 742.7 mm in L_3_, 1,223.8 mm in L_2_, 598.1 mm in L_6_, 1,195.3 mm in L_4_ and 654.8 mm in L_5_, respectively (Table 1). Reports of previous studies explain the significant influence of climatic factors, *viz.*, rainfall, and temperature on soil organic matter and other nutrients, ultimately affecting saffron cultivation (Kamyabi et al., 2014; Mykhailenko et al., 2020). Relative humidity was observed maximum in location L_2_ (60.6%) compared with other locations; however, it was lowest in location L_3_ (38.3%).

The soil pH was lowest in location L_1_ (5.2) followed by L_5_ (5.3), L_6_ (5.6), L_2_ (6.0), L_3_ (6.2), and L_4_ (6.4) (Table 2). Electrical conductivity was observed highest in L_1_; however, lowest in L_2_ and L_6_ locations. According to Ganaie and Singh (2019), slightly alkaline soil with pH in the range of 6.3 to 8.3 and electrical conductivity in the range of 0.09 to 0.30 ds/m is most suitable for increasing saffron productivity. The soil textures of the selected study sites were sandy loam to clay loam. In Iran’s climatic conditions, sandy soil has been reported as a useful soil for raising the size of corms and flowers along with pH levels 6.8 and 7.8 (Mykhailenko et al., 2020). The percentage of soil organic carbon was significantly higher in location L_1_ (1.2%) followed by L_2_ (1.0%), L_3_ (0.9%), and L_4_ (0.8%). The soil of location L_5_ was medium in organic carbon (0.7%) but low at location L_6_ (0.4%). This increase in soil organic matter at high altitudes might be due to the higher input of organic matter and limited decomposition rate by lesser temperature and higher water retention capacity. The available nitrogen was medium in locations L_1_ (269.4 kg/ha), L_3_ (448.4 kg/ha), L_2_ (313.8 kg/ha), and L_4_ (283.5 kg/ha) yet low in locations L_6_ (150.6 kg/ha) and L_5_ (167.7 kg/ha). Across six different locations, the value of available phosphorus was medium in the range at all the locations except for location L_3_. The content of available potassium was very high for location L_1_ (887.5 kg/ha) followed by L_3_ (856.3 kg/ha) and L_2_ (640.8 kg/ha). Available potassium for location L_4_ (439.3 kg/ha) was recorded higher in range, while, L_6_ (229.4 kg/ha) and L_5_ (246.5 kg/ha) locations were medium in potassium availability. This might be due to the variability of temperature and total rainfall in different geographical locations. The soils of most of the selected locations were sandy loam in texture except for locations L_6_, which were sandy clay loam (Table 2).

**TABLE 2:** Variation in soil physicochemical properties at different altitudinal locations.

**Locations**	**pH**	**Electrical conductivity (m mhos/cm)**	**Organic carbon (%)**	**Available N (kg/ha)**	**Available P_2_O_5_ (kg/ha)**	**Available K_2_O (kg/ha)**	**Sand (%)**	**Silt (%)**	**Clay (%)**	**Soil texture**
L_1_	5.2 ± 0.2	0.20 ± 0.05	1.2 ± 0.2	269.4 ± 0.2	17.0 ± 0.5	887.5 ± 0.3	44.3 ± 0.4	32.2 ± 0.4	23.5 ± 0.2	Sandy loam
L_2_	6.0 ± 0.3	0.12 ± 0.01	1.0 ± 0.4	313.8 ± 0.3	13.9 ± 0.7	640.8 ± 0.5	51.1 ± 0.4	34.5 ± 0.5	14.4 ± 0.1	Sandy loam
L_3_	6.2 ± 0.1	0.17 ± 0.02	0.9 ± 0.6	448.4 ± 0.3	8.2 ± 0.4	856.3 ± 0.2	44.8 ± 0.6	31.7 ± 0.2	23.5 ± 0.4	Sandy loam
L_4_	6.4 ± 0.1	0.30 ± 0.25	0.8 ± 0.2	283.5 ± 0.2	16.4 ± 0.3	439.3 ± 0.7	56.1 ± 0.2	30.5 ± 0.4	13.4 ± 0.2	Sandy loam
L_5_	5.3 ± 0.3	0.18 ± 0.06	0.7 ± 0.1	167.7 ± 0.4	16.8 ± 0.3	246.5 ± 0.3	48.9 ± 0.2	30.5 ± 0.2	20.6 ± 0.4	Sandy loam
L_6_	5.6 ± 0.2	0.12 ± 0.04	0.4 ± 0.2	150.6 ± 0.1	19.9 ± 0.4	229.4 ± 0.4	45.9 ± 0.1	32.5 ± 0.9	21.6 ± 0.3	Sandy clay loam

*Data represents ± standard deviation of three samples.*

### Morphological and Productive Traits

The number of flowers/m^2^ and fresh flower yield (kg/ha) was significantly higher during the 2019–2020 compared with the 2018–2019 season (Table 3). The increase in the number of flowers and fresh flower yield during the 2019–2020 was 9.9 and 14.7% compared with the 2018–2019 crop season. The rest of the parameters were not significantly affected by two different cropping years. The better quality corms were produced in the subsequent year, responsible for the higher number of flowers (Bayat et al., 2016; Mykhailenko et al., 2020). Significant differences in weather parameters in given years ensure a direct relationship between studied traits and the environment and weather conditions during both years.

**TABLE 3:** Different altitudinal locations affect growth, yield and yield attributes of saffron.

**Treatment**	**Number of flowers/m^2^**	**Fresh flower yield (kg/ha)**	**Fresh stigma yield (kg/ha)**	**Dry stigma yield (kg/ha)**	**Number of leaves/plant**	**Leaf length (cm)**
**Year**						
Y_1_	26.20^b^	87.84^b^	9.67^b^	1.88^b^	19.66^b^	28.81^b^
Y_2_	28.86^a^	100.71^a^	11.05^a^	2.06^a^	40.38^a^	32.13^a^
**Locations**						
L_1_	25.43^d^	85.26^d^	9.51^d^	1.80^d^	13.16^de^	30.30^d^
L_2_	31.28^b^	117.53^a^	12.45^ab^	2.21^b^	28.16^b^	33.01^c^
L_3_	32.85^a^	111.6^b^	12.51^a^	2.40^a^	42.83^a^	36.65^ab^
L_4_	29.88^c^	68.35^f^	11.80^abc^	2.11^bc^	19.33^c^	28.65e
L_5_	24.21^e^	100.95^c^	9.50^de^	1.72^de^	13.00^def^	37.73^a^
L_6_	21.53^f^	81.98^e^	6.40^f^	1.61^ef^	13.66^d^	16.50^f^

*Means within each column with similar letter are not significantly different at the 5% probability level.*

The different altitudinal locations showed that the results of growth and yield traits were location-specific (Table 3). The number of flowers/m^2^ was significantly higher in L_3_ (32.8); however, it was lowest in L_6_ (21.5). The interaction effect of years and locations highlighted the best performance of saffron when cultivated in location L_3_ in terms of the number of flowers during the second year of crop season (Table 3). Fresh flower yield showed a significant high value in geographical location L_3_ (117.5 kg/ha) followed by L_2_ (111.6 kg/ha), L_4_ (100.9 kg/ha), L_1_ (85.3 kg/ha), and L_5_ (82.0 kg/ha).

The number of flowers and fresh flower yield was significantly higher in a location situated at an altitude of 2,195 m (Suppa, Bharmour, H.P.) as compared with other places (Table 3). It might be because of the favorable environmental conditions, *viz.*, average temperature (28.2°C), total rainfall (742.7 mm) and relative humidity (38.3%). It means that saffron required lower relative humidity to produce a higher yield of spice. Rainfall and temperature (23.0–27.0°C) are the crucial climatic factors controlling the growth and flowering in *Crocus* species (Aghhavani Shajari et al., 2015), and in this study, we have recorded temperature from 20.9 to 27.8°C, which is suitable for the cultivation of a saffron crop in selected locations.

Fresh stigma yield was significantly higher in location L_3_ (12.5 kg/ha) and lowest in L_6_ (6.4 kg/ha). The percentage increase in the L_3_ location was 95.3% as compared with the L_6_ location (Table 3). Dry stigma yield was significantly affected by different altitudinal variations. In location L_3_, significantly higher dry stigma yield was observed compared with other different geographical locations. The percentage increase in L_3_ was 50.0, 41.2, 33.3, 14.3, and 9.1% compared with L_6_, L_5_, L_1_, L_4_, and L_2_ locations, respectively (Table 3). In our studies the average dry stigma yield varied from 1.6 to 2.4 kg/ha, however, in Greece the average yield ranges from 4.0–7.0 kg/ha, Italy from 3.4 to 10.0 kg/ha, in Morocco 2.0–2.5 kg/ha; in Spain 2.5–6.0 kg/ha and in Iran 3.0–5.0 kg/ha (McGimpsey et al., 1997; Gresta et al., 2008). The geographical location of Suppa, Bharmour, H.P. (L_3_) significantly recorded higher dry stigma yield (2.4 kg/ha) as compared with other sites; however, the dry yield of stigma behaved statistically at par with Kapkote, Bageshwar, Uttarakhand (2.2 kg/ha) and Sathli, Bharmour, H.P. (2.1 kg/ha). The high input of organic matter and low decomposition processes is carried out at high altitudes, as temperature and temporal water saturation contributed to increased yield (Davidson and Janssens, 2006).

The lowest dry stigma yield was recorded in Palampur, Himachal Pradesh, as the altitude was relatively low and rainfall was higher as compared with other locations. It might also be related to low pH, organic carbon, available nitrogen, phosphorous, potassium, soil texture (Table 2) and other external environmental factors. Thus, in our studies saffron yield is close to the yield in Morocco and Spain, which showed high environmental adaptability to India’s latitude and produced higher cultivation rates. Specifically, the climatic conditions of these locations were desirable for the production of the saffron corm and other vegetative characteristics. Previous studies reported a 70% increase in saffron flowering when planted in soils with lighter consistency (Aghhavani Shajari et al., 2015); however, an 18% increase was reported in saffron stigma yield when grown in sandy soil as compared with the heavy soil (Khorramdel et al., 2015).

Growth parameters *viz.*, the number of leaves/plant and leaf lengths were significantly affected by different geographical locations (Table 3). A significantly higher number of leaves/plant and leaf length were recorded in L_3_ followed by L_5_, L_2_, L_4_, L_1_, and L_6_. The interaction effect of year and geographical locations was significant on the number of leaves/plant and leaf length. A significantly higher number of leaves and leaf length were observed in location L_3_ during the 2019–2020 crop season. Sand-particles usually cause higher pores, improving the soil’s permeability (Banihabib and Iranpour, 2012). This increases the root growth, improves the production and development of leaves (Fallahi et al., 2017). Panwar et al. (1995) in their study reported efficient cultivation of saffron at the highest altitude, which is most desirable for corm production, suggesting that elevation can play a crucial role. The development of corms is directly dependent on shoots or leaves at appropriate environmental conditions, including temperature, altitude and soil texture for saffron growth (Mykhailenko et al., 2020). Thus, to grow saffron as a valued commodity, one must take measures to achieve ideal conditions for the cultivation and processing of saffron corms in this area.

### Regression and Correlation Analysis

**FIGURE 2:**
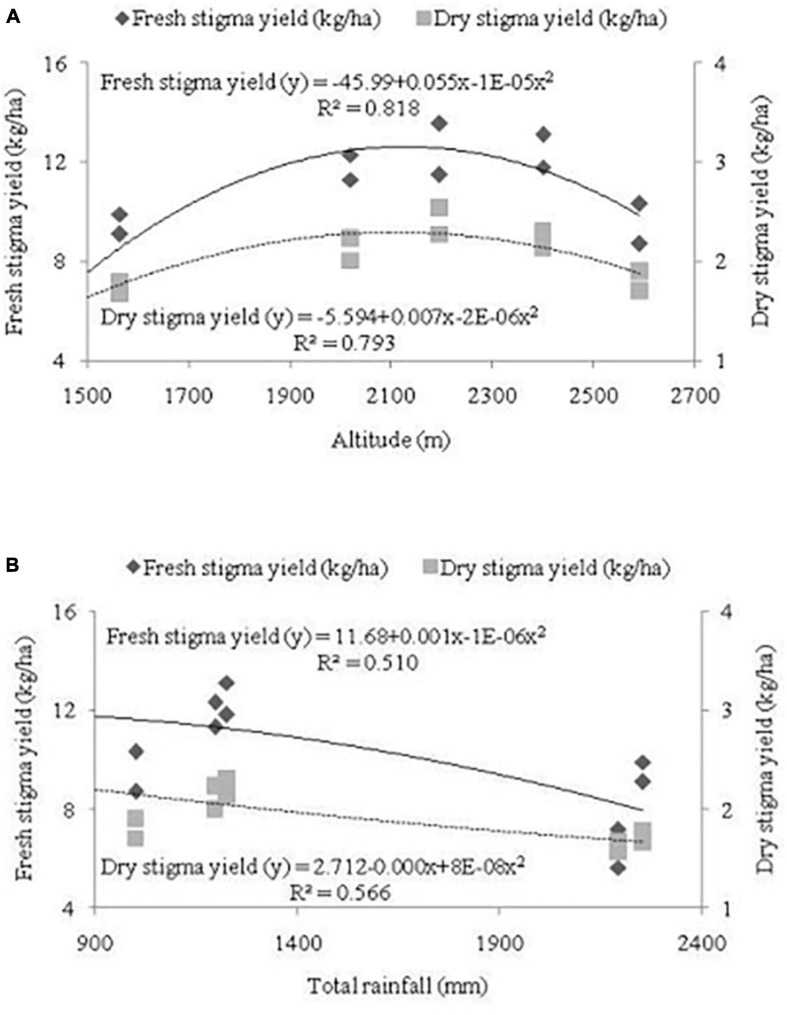
Regression equation between independent variable, **(A)** altitude (m), **(B)** total rainfall and dependent variables, i.e., fresh stigma yield (kg/ha) and dry stigma yield (kg/ha). Altitude and rainfall are represented in the primary X-axis. Fresh stigma yield and dry stigma yield are presented in the primary Y-axis and secondary Y-axis, respectively.

Regression equations have been developed between independent variables, *viz.*, altitude and total rainfall, and dependent variables, *viz*., fresh stigma yield and dry stigma yield (Figure 2). In the current study, fresh stigma yield and dry stigma yield increased with the corresponding increase in altitude (Figure 2A) and was found highest in location L_3_ at altitude 2,195 m. Thus, a strong relationship was formed for different altitude with fresh stigma yield and dry stigma yield with an equation of y = −45.99 + 0.055x-1E-05x^2^ (*R^2^* = 0.818; *P* ≤ *0.01*) and y = −5.594 + 0.007x-2E-06x^2^ (*R^2^* = 0.793; *P* ≤ *0.01*), respectively (Figure 2). Total rainfall, fresh stigma yield and dry stigma yield enhanced with a decrease in altitude (Figure 2B). Therefore, a strong relationship was established by total rainfall with fresh stigma yield; y = 11.68 + 0.001x-1E-06x^2^ (*R^2^* = 0.510; *P* ≤ *0.01*) and dry stigma yield; y = 2.712-0.000x + 8E-06x^2^ (*R^2^* = 0.566; *P* ≤ *0.01*). The current study revealed that with the increase in altitude, fresh and dry stigma yield increases; however, with the increase in rainfall, yield attributes decrease (Figure 2).

**FIGURE 3:**
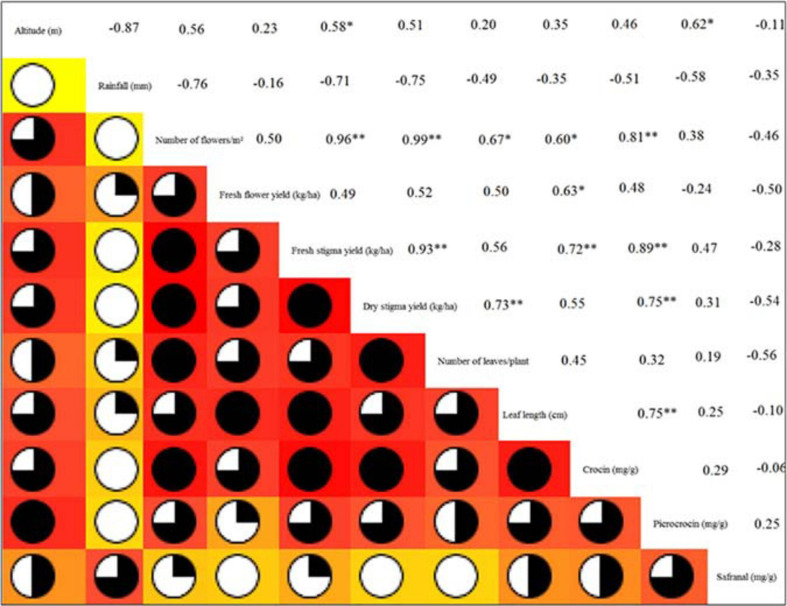
Correlation matrix between dependent and independent variables. ^∗^ and ^∗∗^indicate that the corresponding values are significant at *P* = *0.05* and *P* = *0.01*, respectively.

Yield attributes, genotype and phenotypic correlation were measured to study the relation between saffron yields (Figure 3). Marker compound safranal showed a negative correlation with all the dependent and independent variables. Picrocrocin reported a higher positive correlation to altitude (*r* = 0.62) at a 5% significance level; however, rainfall (*r* = −0.58) and fresh flower yield (*r* = −0.24) showed a negative correlation. The compound crocin reported higher positive correlation with leaf length (*r* = 0.75), dry stigma yield (*r* = 0.75), fresh stigma yield (*r* = 0.89) and number of flowers (*r* = 0.81) at a 1% significance level and showed positive correlation with other variables. Saffron leaf length and leaf number have positive correlations for all characteristics studied except for rainfall. Dry stigma yield had the highest correlation with the number of flowers (*r* = 0.99) and fresh stigma yield (*r* = 0.93) at a 1% significance level. Dry stigma yield and fresh stigma yield have created a strong correlation with the increase in altitude and fresh flower yield, while they are negatively correlated with total rainfall. Fresh stigma yield recorded a substantially greater positive correlation with the number of flowers (*r* = 0.96) at a 1% significance level and altitude (*r* = 0.58) at a 5% significance level. Fresh flower yield reported positive correlation with number of flowers (*r* = 0.50) and altitude (*r* = 0.23), while, showed negative correlation with temperature (*r* = −0.16). A positive correlation was reported for the number of flowers with an increase in altitude. Total rainfall produced a negative correlation with yield attributes, yield and marker compounds of the saffron crop. The results indicated that selecting these traits is useful to improve the dry stigma yield of saffron (Figure 3). Genetic associations are more significant in correlation studies than phenotypic correlations due to removing environmental effects in calculating genetic correlation coefficients (Singh et al., 2005). Our study indicates that saffron yield attributes and the yield itself correlate greatly with altitude. Hence, optimum environmental conditions improve the yield attributes to produce a higher saffron yield (Figure 3).

### Quality Analysis

#### Principal Component Analysis

**FIGURE 4:**
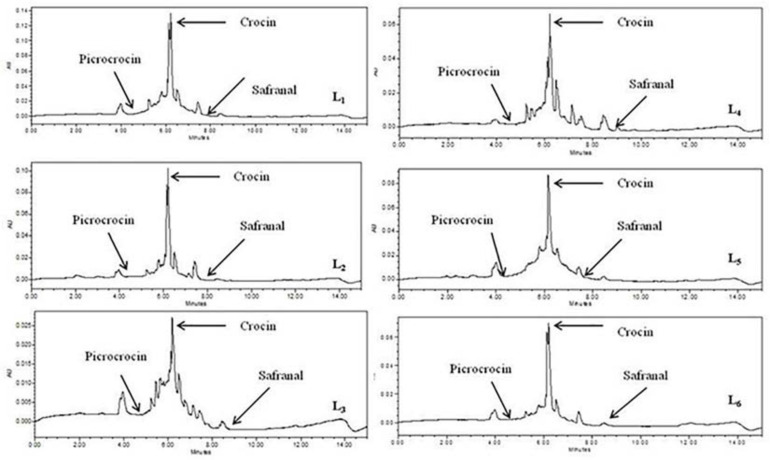
Chromatogram of marker compounds of saffron in different environmental locations.

**FIGURE 5:**
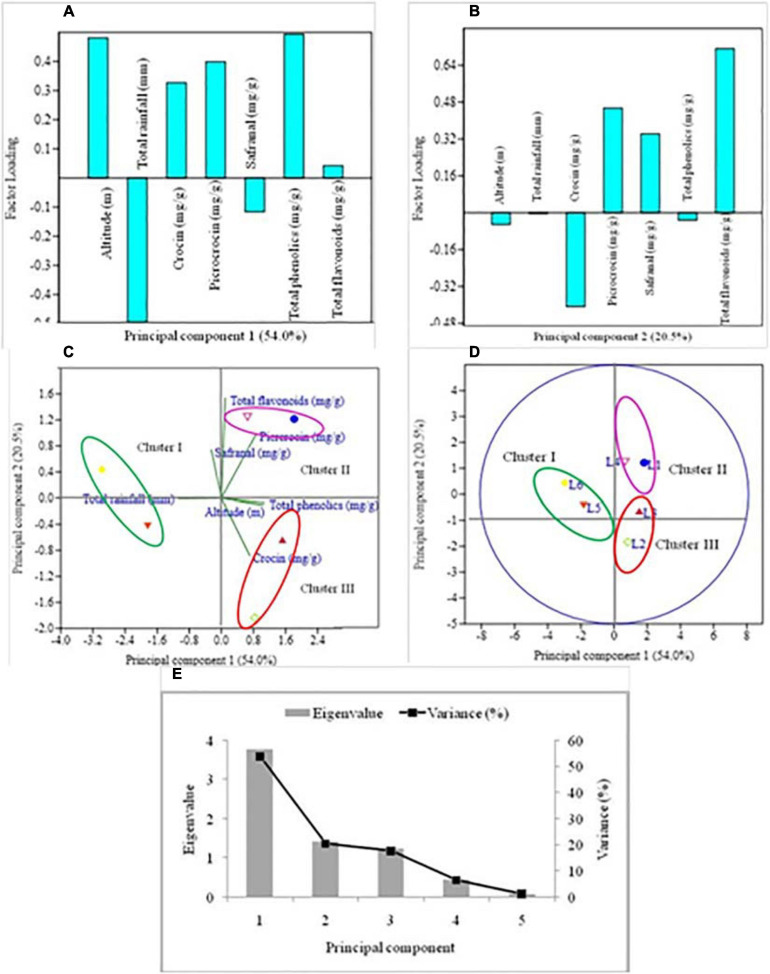
The multivariate analysis of the mean value of independent variables, *viz*., altitude and total rainfall and secondary metabolites were conducted through principal component analysis. PC-1 and PC-2 jointly explained the variations of 74.5% **(A–E)**. **(A)** represent variables of loading plot PC-1, **(B)** variables of loading plot PC-2, **(C)** variables presented as vectors in the space of the PCA, **(D)** loading scores of the treatments with PC-1 and PC-2, and **(E)** represent eigenvalue.

In this trial, we used PCA to analyze and acknowledge the relationship between independent variables (altitude and rainfall) with secondary metabolites (marker compounds, total phenolics and flavonoids) and how they vary according to the different altitudinal locations. The composition of secondary metabolites was taken as dependent variables. Three marker compounds, total phenolics and flavonoids of dry stigma samples from six different geographical areas were subjected to PCA for analyzing compositional variation. The chromatogram of marker compounds of different geographical locations has been presented in Figure 4. The correlation coefficient of secondary metabolites in PCA is determined by the cosine of the angle between their vectors (Dehghani et al., 2006). PCA plots jointly explained the variance of 74.5% (Figure 5). PC-1 explained 54.0% of the total variation, accounting for the positive contribution of altitude, crocin, picrocrocin, total phenolics and flavonoids; however, the negative contribution of total rainfall and safranal compound (Figure 5A). PC-2 explained 20.5% of the total variance, clearly distinguishing altitude, total precipitation, crocin and total phenolics in negative contribution with picrocrocin, safranal and total flavonoids in positive contribution (Figure 5B). Figures 5C,D showed the distribution of variables and treatments, respectively.

In the present study, three distinct clusters were observed in the score plot (Figures 5C,D). Cluster I from Langha, Palampur, Himachal Pradesh (L_5_), explained a higher concentration of crocin (48.8 mg/g), picrocrocin (22.8 mg/g), safranal (3.8 mg/g), and total phenolics (5.9 mg/g); however, the total flavonoid count (4.9 mg/g) was higher in CSIR-IHBT, Palampur, Himachal Pradesh. In cluster II location of Sathli, Bharmour, Himachal Pradesh (L_4_) explained the higher concentration of crocin (52.5 mg/g) and total flavonoid (mg/g); however, geographical location L_1_ produced a higher concentration of picrocrocin (36.5 mg/g) and total phenolics (7.1 mg/g). Cluster III included two geographical locations (L_2_ and L_3_) showing a higher concentration of all the secondary metabolites in location L_3_ (57.8 mg/g), except for crocin (57.8 mg/g) which was higher in the L_2_ location (Table 4). This study also showed that the first three PCs with eigenvalues 3.8, 1.4, and 1.2 were most informative accounting for approximately 92.2% of the overall variance for all variables (Figure 5E). The saffron’s quality is a difficult parameter because it depends on several factors, in particular environmental conditions, including altitude, temperature, precipitations that affect the anatomy and quantity *viz.*, crocin, picrocrocin and safranal (Cardone et al., 2019). Climatic factors, *viz.*, altitude might also influence the quality of saffron spice, especially crocin (Lage and Cantrell, 2009). Fertile soils and a favorable climate distinguish these regions.

**TABLE 4:** Cluster variability in secondary metabolites of saffron affected by altitudinal locations.

**Sr. No.**	**Compounds**	**Cluster I**	**Cluster II**	**Cluster III**
1	Crocin	25.1–48.8	41.0–52.5	53.6–57.8
2	Picrocrocin	18.8–22.8	34.6–36.5	25.6–26.1
3	Safranal	2.4–3.8	3.4	0.8–2.5
4	Total phenolics	5.4–5.9	6.3–7.1	6.5–7.0
5	Total flavonoids	4.5–5.1	5.1–5.5	3.4–5.2

Factors affecting the accretion of compounds in plants used as medicinal and pharmaceutical raw materials and food additives are dependent on altitude, temperature, soil type, irrigation cycles, plant quality, and harvest times of saffron crop (Mykhailenko et al., 2020). The content of crocin was significantly higher in location Sathli, Bharmour, H.P. (L_4_), and picrocrocin was markedly higher in Kapkote, Bageshwar, Uttarakhand (L_2_); however, safranal was higher in the sample from CSIR-IHBT, Palampur, H.P. (Figures 4, 5). Increased crocin and picrocrocin at high altitudes might be due to total rainfall, air temperature, solar radiation, and soil characteristics, significantly affecting the accumulation of marker compounds in plants (Mykhailenko et al., 2020). The drying process is also a vital course ensuring a product’s quality (Maghsoodi et al., 2012). Previous studies indicated that with the increase in altitude, the crocin content increased. Thus, agronomical and climatic factors affect the quality of saffron (Lage and Cantrell, 2009). Total phenolics and flavonoids were significantly higher in high altitude locations as compared with lower altitude. The annual precipitation in comparison with high altitude regions was higher at low altitudes. Therefore, it was clear that different environmental conditions influenced the alteration of secondary metabolites of saffron.

## Conclusion

This study revealed significant differences in different altitudinal locations for all studied traits for the saffron crop. Thus, the accurate selections of the geographical locations are considered important and essential factors. The yield attributes, *viz.*, the flower number/m^2^, fresh flower yield, and dry stigma yield were significantly higher in the second year at the geographical location of Suppa, Bharmour, H.P. (L_3_), which confirmed that these traits are significantly influenced by environmental factors. Results of independent variables, genotypic and phenotypic coefficients of variation revealed a positive correlation for saffron yield with most characteristics. Thus, it was concluded that saffron could successfully be cultivated in the hilly regions of Himachal Pradesh. The studies suggest a need to have more research work on multiple sites in the same geographical locations with other factors that influence the quality of saffron production with altitude. Moreover, for each geographical location, there is a need to study the effect of saffron quality with meteorological data.

## Data Availability Statement

The raw data supporting the conclusions of this article will be made available by the authors, without undue reservation.

## Author Contributions

DK: experiment execution, data collection, soil and quality analysis, literature search, and manuscript writing. MT: data collection, statistical analysis and data presentation, literature search, and manuscript writing. RJ: quality analysis. AK: identification of sites through MAXENT model. RK: develop the idea, overall guidance, and manuscript editing. All authors contributed to the article and approved the submitted version.

## Conflict of Interest

The authors declare that the research was conducted in the absence of any commercial or financial relationships that could be construed as a potential conflict of interest.
