# Supplementary material for: Agro-Climatic Suitability Evaluation for Saffron Production in Areas of Western Himalaya
Source: Front Plant Sci. 2021 Mar 15;12:657819. doi: 10.3389/fpls.2021.657819 (PMC8005729; doi:10.3389/fpls.2021.657819)
Supplement: Supplementary file 1 [file Table_1.DOCX]

| Code | Location | | | Geographical coordinates | | Altitude  (m) | Average temperature  (°C) | | Total rainfall  (mm) | Relative humidity (%) |
| --- | --- | --- | --- | --- | --- | --- | --- | --- | --- | --- |
| Site | Site | District | State | Latitude | Longitude |  | Max. | Min. |  |  |
| L_1_ | Moorang | Kinnaur | Himachal Pradesh | 31°36’10” | 78°27’04” | 2591 | 20.9 | 8.5 | 998.9 | 49.6 |
| L_2_ | Kapkote | Bageshwar | Uttarakhand | 30°05’05” | 79°53’58” | 2400 | 23.9 | 8.6 | 1223.8 | 60.6 |
| L_3_ | Suppa, Bharmour | Chamba | Himachal Pradesh | 32°26’47” | 76°34’14” | 2195 | 28.2 | 16.5 | 742.7 | 38.3 |
| L_4_ | Sathli, Bharmour | Chamba | Himachal Pradesh | 32°26’47” | 76°34’14” | 2019 | 27.5 | 17.1 | 1195.3 | 42.7 |
| L_5_ | Langha, Palampur | Kangra | Himachal Pradesh | 32°07’49’’ | 76°33’54” | 1565 | 26.2 | 15.7 | 2254.8 | 46.2 |
| L_6_ | CSIR-IHBT Palampur | Kangra | Himachal Pradesh | 32°06’29’’ | 76°33’35” | 1472 | 27.8 | 17.7 | 2193.1 | 46.8 |

**Table 1.** Location, geographical and climatic characteristics of the study area of saffron crop

**Table 2.** Variation in soil physico-chemical properties at different altitudinal locations

| Locations | pH | Electrical conductivity  (m mhos/cm) | Organic carbon  (%) | Available N  (kg/ha) | Available P_2_O_5_  (kg/ha) | Available K_2_O  (kg/ha) | Sand  (%) | Silt  (%) | Clay  (%) | Soil texture |
| --- | --- | --- | --- | --- | --- | --- | --- | --- | --- | --- |
| L_1_ | 5.2±0.2 | 0.20±0.05 | 1.2±0.2 | 269.4±0.2 | 17.0±0.5 | 887.5±0.3 | 44.3±0.4 | 32.2±0.4 | 23.5±0.2 | Sandy loam |
| L_2_ | 6.0±0.3 | 0.12±0.01 | 1.0±0.4 | 313.8±0.3 | 13.9±0.7 | 640.8±0.5 | 51.1±0.4 | 34.5±0.5 | 14.4±0.1 | Sandy loam |
| L_3_ | 6.2±0.1 | 0.17±0.02 | 0.9±0.6 | 448.4±0.3 | 8.2±0.4 | 856.3±0.2 | 44.8±0.6 | 31.7±0.2 | 23.5±0.4 | Sandy loam |
| L_4_ | 6.4±0.1 | 0.30±0.25 | 0.8±0.2 | 283.5±0.2 | 16.4±0.3 | 439.3±0.7 | 56.1±0.2 | 30.5±0.4 | 13.4±0.2 | Sandy loam |
| L_5_ | 5.3±0.3 | 0.18±0.06 | 0 .7±0.1 | 167.7±0.4 | 16.8±0.3 | 246.5±0.3 | 48.9±0.2 | 30.5±0.2 | 20.6±0.4 | Sandy loam |
| L_6_ | 5.6±0.2 | 0.12±0.04 | 0.4±0.2 | 150.6±0.1 | 19.9±0.4 | 229.4±0.4 | 45.9±0.1 | 32.5±0.9 | 21.6±0.3 | Sandy clay loam |

Note: Data represents ± standard deviation of three samples

**Table 3.** Different altitudinal locations affect growth, yield and yield attributes of saffron

| Treatment | Number of flowers/m^2^ | Fresh flower yield  (kg/ha) | Fresh stigma yield  (kg/ha) | Dry stigma yield  (kg/ha) | Number of leaves/plant | Leaf length  (cm) |
| --- | --- | --- | --- | --- | --- | --- |
| Year |  |  |  |  |  |  |
| Y_1_ | 26.20^b^ | 87.84^b^ | 9.67^b^ | 1.88^b^ | 19.66^b^ | 28.81^b^ |
| Y_2_ | 28.86^a^ | 100.71^a^ | 11.05^a^ | 2.06^a^ | 40.38^a^ | 32.13^a^ |
| Locations |  |  |  |  |  |  |
| L_1_ | 25.43^d^ | 85.26^d^ | 9.51^d^ | 1.80^d^ | 13.16^de^ | 30.30^d^ |
| L_2_ | 31.28^b^ | 117.53^a^ | 12.45^ab^ | 2.21^b^ | 28.16^b^ | 33.01^c^ |
| L_3_ | 32.85^a^ | 111.6^b^ | 12.51^a^ | 2.40^a^ | 42.83^a^ | 36.65^ab^ |
| L_4_ | 29.88^c^ | 68.35^f^ | 11.80^abc^ | 2.11^bc^ | 19.33^c^ | 28.65e |
| L_5_ | 24.21^e^ | 100.95^c^ | 9.50^de^ | 1.72^de^ | 13.00^def^ | 37.73^a^ |
| L_6_ | 21.53^f^ | 81.98^e^ | 6.40^f^ | 1.61^ef^ | 13.66^d^ | 16.50^f^ |

Note: Means within each column with similar letter are not significantly different at the 5% probability level.

**Table 4.** Cluster variability in secondary metabolites of saffron affected by altitudinal locations

| Sr. No. | Compounds | Cluster I | Cluster II | Cluster III |
| --- | --- | --- | --- | --- |
| 1 | Crocin | 25.1-48.8 | 41.0-52.5 | 53.6-57.8 |
| 2 | Picrocrocin | 18.8-22.8 | 34.6-36.5 | 25.6-26.1 |
| 3 | Safranal | 2.4-3.8 | 3.4 | 0.8-2.5 |
| 4 | Total phenolics | 5.4-5.9 | 6.3-7.1 | 6.5-7.0 |
| 5 | Total flavonoids | 4.5-5.1 | 5.1-5.5 | 3.4-5.2 |
