# Supplementary figures and images for: Agro-Climatic Suitability Evaluation for Saffron Production in Areas of Western Himalaya
Source: Front Plant Sci. 2021 Mar 15;12:657819. doi: 10.3389/fpls.2021.657819 (PMC8005729; doi:10.3389/fpls.2021.657819)

**Graphical abstract**


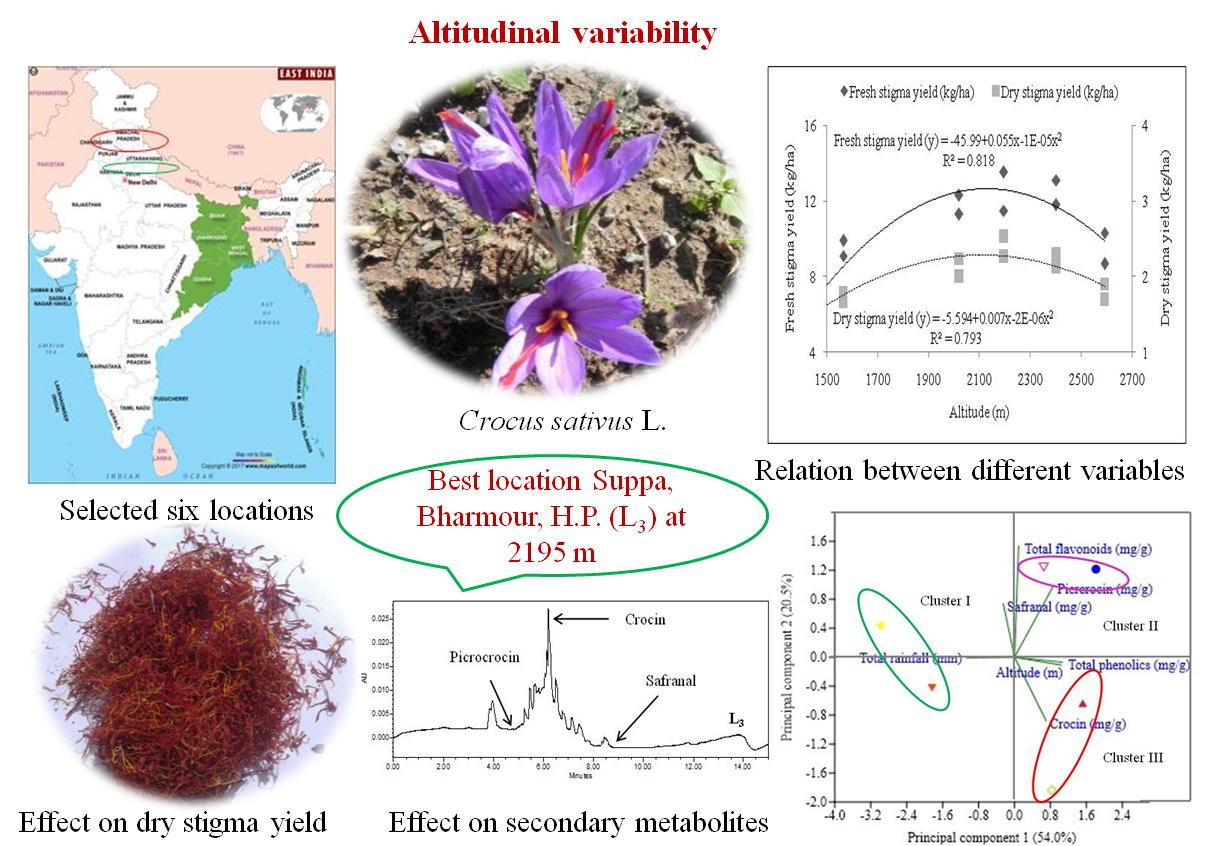

Supplement: Supplementary file 2 [file Data_Sheet_1.docx]
